# Factors associated with medical students’ career choice in different specialties: a multiple cross-sectional questionnaire study at a German medical school

**DOI:** 10.1186/s12909-024-05751-1

**Published:** 2024-07-24

**Authors:** Tobias Leutritz, Maike Krauthausen, Anne Simmenroth, Sarah König

**Affiliations:** 1 Institute of Medical Teaching and Medical Education Research, University Hospital Würzburg, Josef-Schneider-Straße 2/D6, Würzburg, 97080 Germany; 2 Institute of General Practice, University Hospital Würzburg, Josef-Schneider-Straße 2/D7, 97080 Würzburg, Germany

**Keywords:** Undergraduate medical education, Career choice, Specialisation, Personality traits, Workstyle preferences, Admission characteristics, Motives and role models, Biography

## Abstract

**Background:**

Given the shortage and unequal distribution of physicians across specialties, we aimed to evaluate factors associated with medical students’ career choices, including background, personality traits, educational experience, personal interests, lifestyle considerations, and the awareness of work requirements.

**Methods:**

We conducted multiple cross-sectional surveys of students; a 159-item online questionnaire was designed and students from three different stages of the six-year medical degree course (outset, clinical phase, and on graduation) were invited to complete the survey. Data were collected between May 2021 and April 2023.

**Results:**

The questionnaire was sent to 1406 students, of whom 683 replied (49%); 481 respondents were female (70%). The top specialty choices across the respondents were internal medicine, surgery, and general practice, with anaesthesiology, paediatric and adolescent medicine (ranging 11–15%), and obstetrics and gynaecology also receiving interest, with 6% undecided. In particular, female students lost interest in surgery during the course of study in favour of the other options. The choice of general practice was associated with more vocational training, prior positive experiences with the specialty, and lower grades in the university entry examination. Clinical clerkships in a specific (freely chosen) specialty aligned with career choice, while the final practical year did not have an impact on career decision-making. All students highly desired regulated working hours and work-life-balance; however, students choosing surgery rated these items as less important. Willingness to work in a hospital environment was highly associated with choosing anaesthesiology and surgery, whereas rural areas and practices were associated with general practice. Higher scores at agreeableness were associated with choosing paediatric and adolescent medicine by more female students, whereas lower neuroticism values were associated with the choice of anaesthesiology.

**Conclusions:**

The results highlight the intricate nature of decision-making and shed light on various aspects that contribute to the process of selecting a specialty. By identifying and addressing influencing factors, we can develop targeted interventions and policies to enhance diversity and distribution across medical specialisations and to aim for high-quality and equitable healthcare that matches the specific needs of both individuals and the population as a whole.

**Graphical Abstract:**

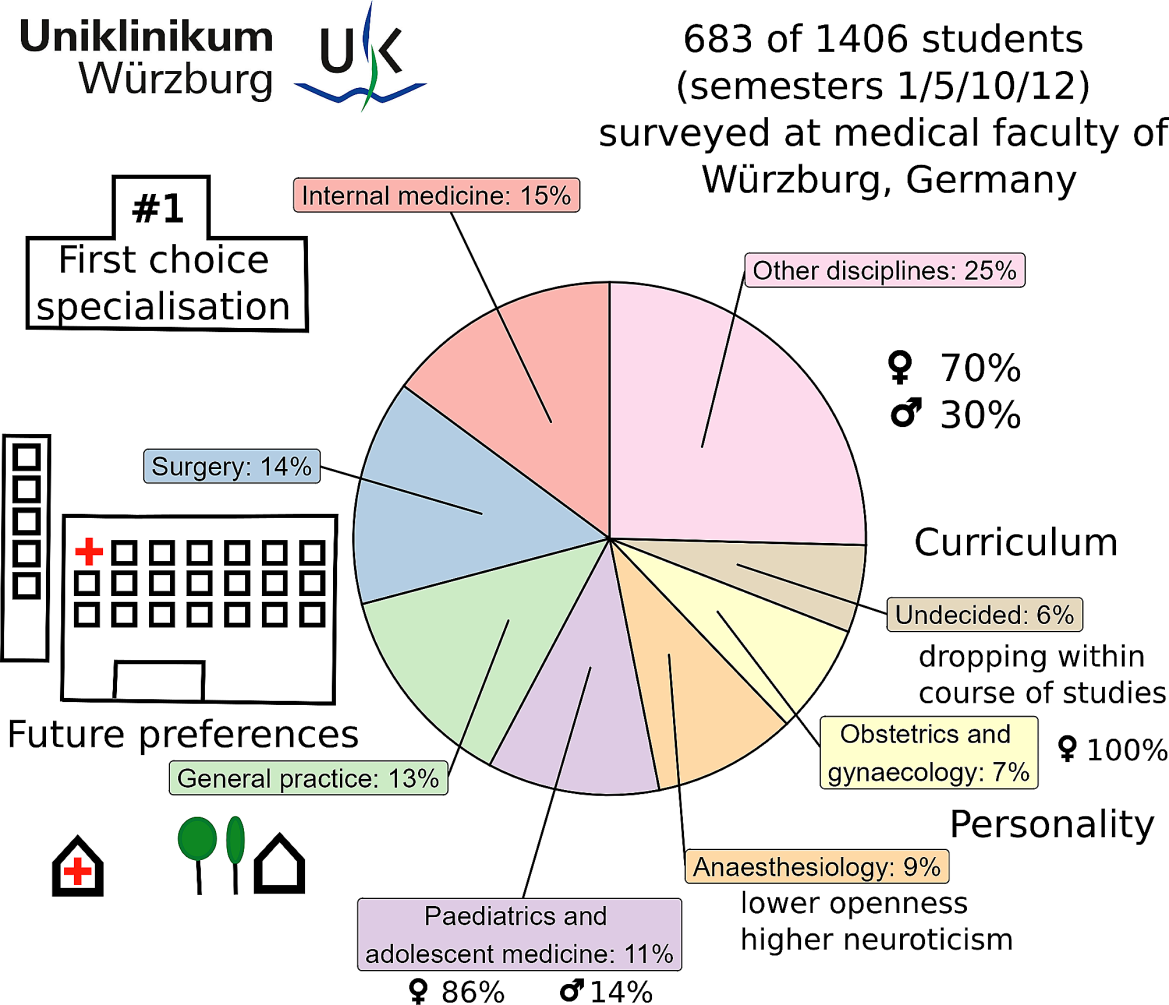

**Supplementary Information:**

The online version contains supplementary material available at 10.1186/s12909-024-05751-1.

## Background

### An increasing shortage of physicians

Germany, like many other developed countries, is facing a shortage of physicians, not only in primary care, but also in the hospital sector. The medical profession is ageing in reflection of society as a whole. The burden of multiple and chronic diseases requires more medical attention and complex care coordination, which in turn puts pressure on the healthcare system [1]. The shortage of physicians has resulted in an increased workload for existing doctors and, in the worst-case scenario, longer waiting times for patients and reduced access to medical care in rural and underserved regions [2, 3]. Patients often face the hurdle of long distances to travel, resulting in delays in care and compromised health outcomes [4]. According to a study by The Associations of Statutory Health Insurance Physicians (Kassenärztliche Vereinigungen; ASHIPs), the current shortage of practicing physicians in Germany is estimated to be around 4,100 full-time equivalents in general practice, and approximately 1,000 in specialist care as of 2021 [5]. If no measures are taken, these numbers are expected to increase in the coming years.

The causes of physician shortage are multifactorial. One of the main factors is the capacity of medical schools in Germany to produce medical graduates. In addition, there is a generational shift, as a significant number of physicians approach retirement age [6]. Other factors include high workload and a stressful work environment, which can lead to medical leave and burnout. This, in turn, may result in a desire for part-time work, early retirement, and in the worst-case scenario, leaving clinical practice altogether [7, 8]. Finally, to improve work-life balance, many doctors nowadays choose not to work full-time voluntarily. As a result, statistically, it takes 1.2 young doctors to replace one retiring physician [9].

Various measures have been proposed to address the shortage of physicians in Germany. Increasing the number of medical graduates is a logical step to tackle the growing demand [10]. In addition, strategies are being developed to improve working conditions and reduce administrative workloads, in order to enhance the value of the medical profession [11]. Further actions include financial incentives for physicians, and investing in technology and infrastructure to improve patient access to care and to ease the provision of healthcare, particularly in rural areas [12, 13]. However, implementing these measures requires a concerted effort from various stakeholders, including policymakers, medical associations, and healthcare providers.

### Relative scarcity of physicians across different specialties and regions

In addition to the absolute shortage of physicians, there is the issue of relative scarcity, which describes an imbalance in the distribution of physicians across disciplines and geographical regions. There is a potential mismatch between the interests of graduates and trainees, and the future needs of the population [14]. As such, the distribution of physicians among different (sub-)disciplines in medicine needs to be reviewed to ensure that all the needs in the healthcare system are covered [15]. Nevertheless, this requires qualified and suitable doctors in the first place. So far, the new system of needs-based planning has already partially improved the situation for general practitioners [16]. However, it remains crucial to highlight that this imbalance in both professional and geographical distribution poses a significant challenge in all fields of outpatient and inpatient care. Furthermore, shortages in certain medical specialties can lead to an over-reliance on specialists and suboptimal care coordination, further exacerbating the problem [17].

### Young doctors’ decisions regarding their career choices and job opportunities

Understanding the factors that influence the choice of specialty by students can be instrumental in attracting and retaining physicians in underserved fields, as well as addressing the aforementioned workforce shortage and uneven distribution [18]. However, the decision to pursue a specific medical career is complex and influenced by a wide range of elements. These include various personal, social, and professional factors such as gender, origin, personal characteristics, and interests including lifestyle and work preferences; the perceived needs of society; and exposure to the medical curriculum encompassing clinical experiences [19–21].

Admission to medical school in Germany is highly competitive owing to the capacity being regulated centrally. The allocation of places to study medicine ab initio is coordinated by the Foundation for University Admission (Stiftung Hochschulstart). The two main criteria for admission into medical school comprise the grade of the German university entrance qualification (or equivalent school-leaving certificate), as well as the score attained in the Test for Medical Degree Courses [22]. Additionally, some German federal states introduced a quota attempting to increase the numbers of rural doctors (‘Landarztquote’) by providing an additional pathway to medical school, contractually obliging students to pursue a career in general practice on graduation [23, 24].

### Research questions

In a sample of multiple cross-sectional surveys conducted at the Faculty of Medicine, University of Würzburg (Germany), we investigated factors that influence career choices made by students. The objective of this study was to examine the following research theses:

1. Student preferences of specialty change during the progression of their medical studies and are influenced by course-related aspects.
2. Admission characteristics, motives to study medicine, and role models are associated with career choice.
3. Ideas concerning future practice/working conditions are perceived differently depending on choice of specialty.
4. Personality traits (Big Five) and gender are also associated with the preference towards specific specialties.

## Materials and methods

### Questionnaire

The authors designed the 159-item questionnaire, based on previous studies, to which we refer in the following. In the first part, general data were collected as individual biographic and demographic items, school leaving certificate grades, motives, and the influence of role models in choosing to study medicine [25]. Of note, the school leaving certificate passing grade in Germany ranges from 1.0 (best) to 4.0 (worst). To study medicine, the absolute point score is taken into account, which leads to fictitious grades of < 1.0 for point scores > 822. Personality traits were assessed using the 21-item short version of the Big Five Inventory (BFI-K) [26], measuring the widely known five-factor model for describing human personality [27]. The BFI-K includes five broad dimensions of personality: openness to experience, conscientiousness, extraversion, agreeableness, and neuroticism. Finally, participants were asked to indicate their current first-choice specialty, to rate specific aspects related to their study of medicine (e.g., exam grades), and to provide ideas regarding their future practice of medicine [28].

The questionnaire utilized a combination of single or multiple-choice options, binary scales, five or seven-step Likert scales, and semantic differentials with a neutral option. It was mandatory for participants to respond to all items in order to complete the survey. However, if they did not wish to or were unable to provide a specific response, the option “not specified” could be selected. Voluntary free-text response questions were included to allow participants to add additional information.

As the questionnaire was designed in a cross-sectional manner, we incorporated filters to display only relevant questions, such as the number of vocational training courses. Additional questions regarding students working as student assistants or embarking on working towards a doctoral thesis were only applicable to students beyond their second year of studies, which reduced the total number of responses. Therefore, the precise sample size is indicated in the figures.

An excerpt of questions relevant to this study is added as supplement.

### Participants and study design

The prospective cross-sectional study was conducted at the Faculty of Medicine, University of Würzburg, Germany. Würzburg follows a standard six-year curriculum, which includes two preclinical years of teaching, three clinical years, and one practical year of work-place based training. During the clinical years, students must choose four one-month clinical clerkships, including a mandatory clerkship in general practice. After the final state examination, graduates apply for their medical license (Approbation) and then choose a specialty for postgraduate training.

Students were requested to fill in the questionnaire using EvaSys^®^ (Lüneburg, Germany), an online survey service, and links were distributed via e-mail to different cohorts of undergraduate students between May 2021 and April 2023. The survey was managed electronically, with six reminders (on average) being sent out to non-responding participants at one to two-week intervals. To complete the quantitative study, the questionnaire was distributed to different groups, aiming to pool the data: first year = start of the degree course (three surveys), third year (two surveys) and fifth year (one survey) both during the clinical phase. The survey was completed after the final practical year and just before the final state examination = on graduation (three surveys).

### Statistics

**Fig. 1:**
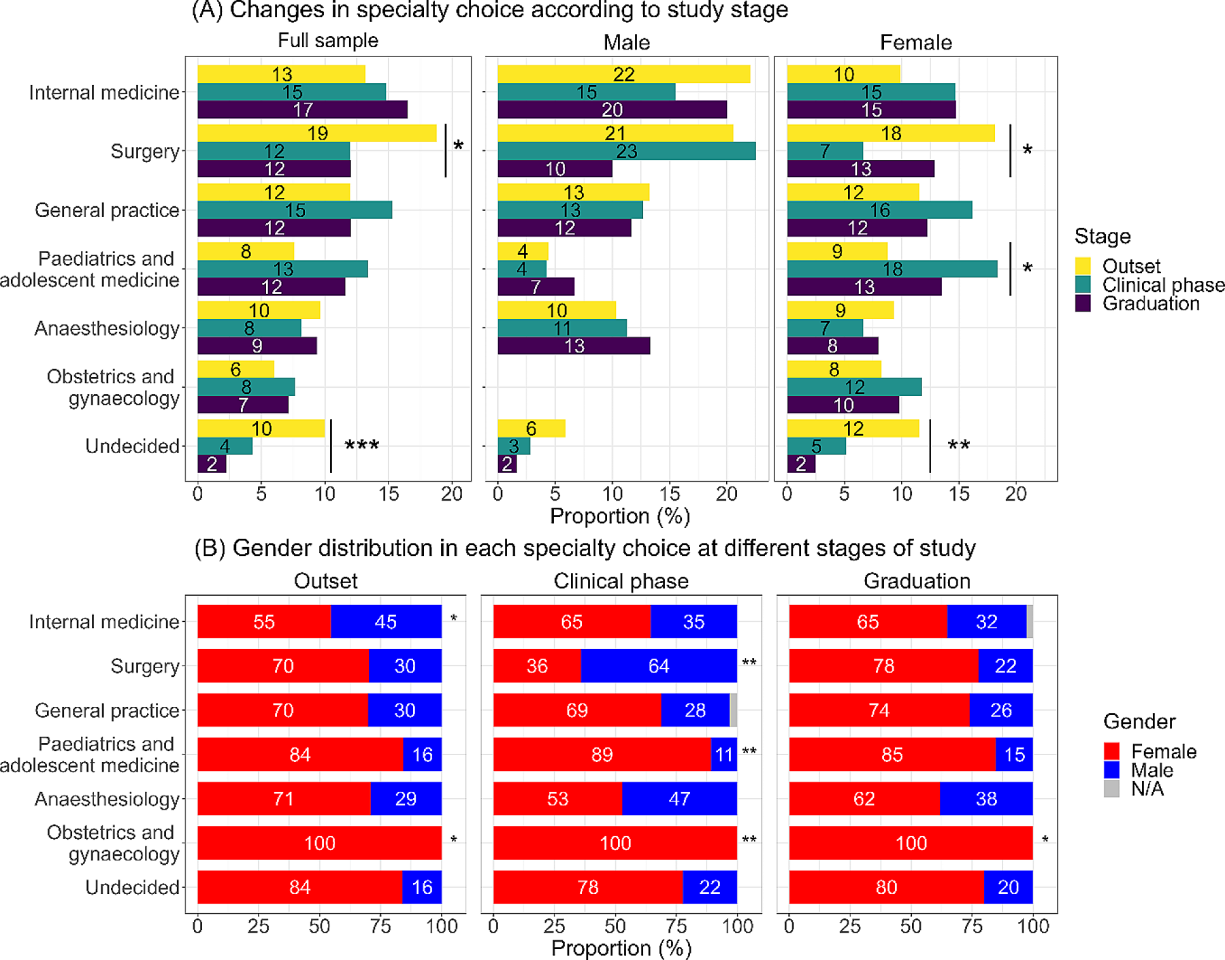
Relative distribution for first choice of the specialisation field in descending order of occurrence including the option “undecided”. *Changes in specialty choice according to study stage for the full sample as well as for male and female subgroups* (**A**) and gender distribution in each specialty choice at different stages of study (outset, clinical phase, and on graduation) (**B**). Asterisks (*: *p* < 0.05, **: *p* < 0.01, ***: *p* < 0.001) mark significant ANOVA results for distribution depending on (A) study phase and (B) field of specialisation

In order to ensure sufficient data for analysis, only specialties that had a selection rate of at least 5% on average were included in the study. Statistical analyses were performed using R 4.3.1 [29], and logistic regressions between the first choice and various factors were performed using the R package mfx [30]. Analysis of variance (ANOVA) was conducted to examine the choice of specialty across different study stages and between different genders (Fig. 1).

**Fig. 2:**
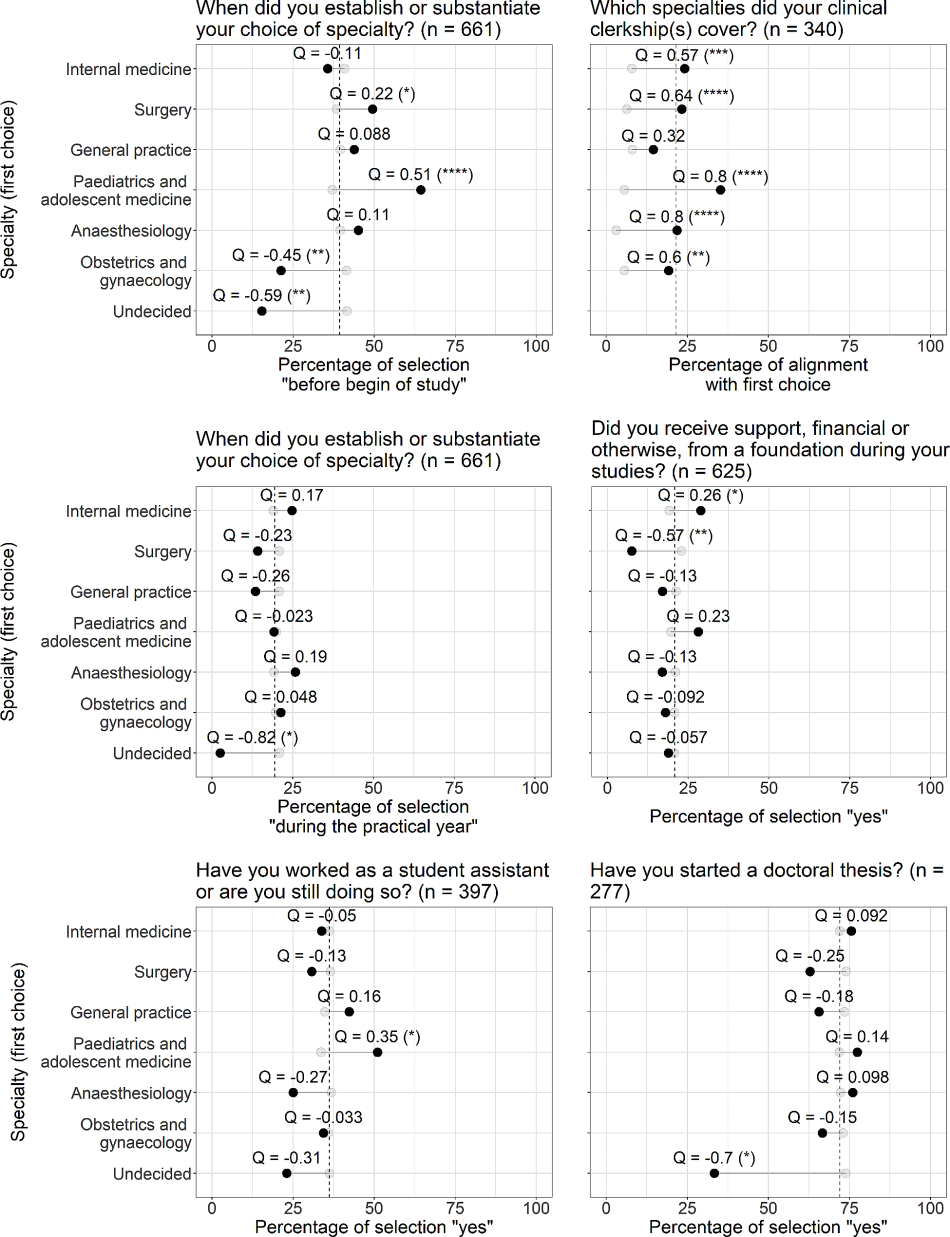
Portrayal of student preferences during their medical studies and course-related characteristics. Mean values for groups having chosen (black circle) vs. not chosen (grey circle) the respective specialty as first choice, as well as overall mean value for the whole sample (dashed black line). Normalized Odds Ratios (Yule’s Q) quantify the strength of association (ranging from − 1 to 1, where 0 indicates no association). Significant results are indicated as * (*p* < 0.05), ** (*p* < 0.01), *** (*p* < 0.001), and **** (*p* < 0.0001)

**Fig. 3:**
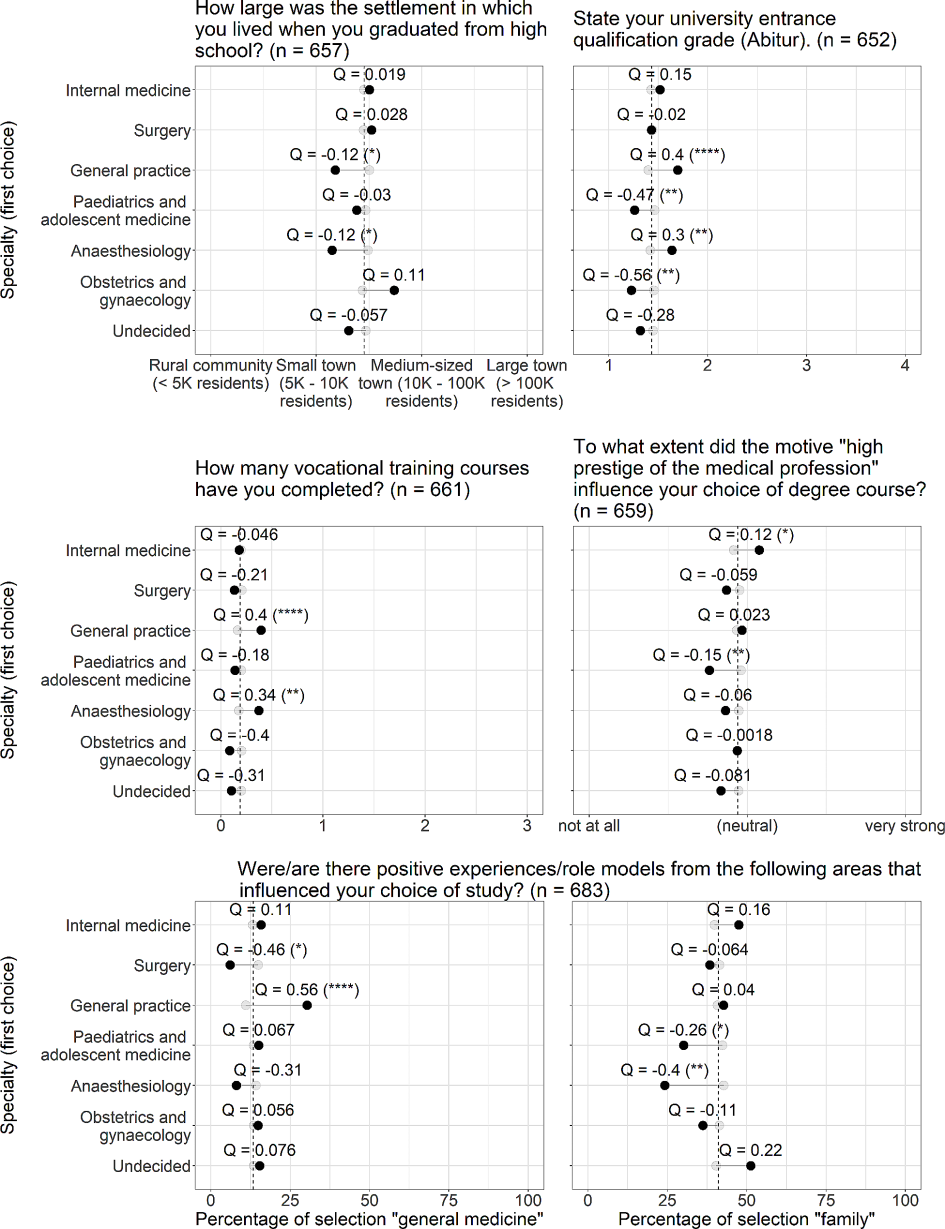
Admission characteristics, motives, and role models influencing the choice to study medicine and choice of specialty. The school leaving certificate passing grade in Germany ranges from 1.0 (best) to 4.0 (worst). A number of vocational training placements prior to medical school exceeding two was recoded as three (only two incidences). For a detailed description of the lollipop plot, see Fig. 2

**Fig. 4:**
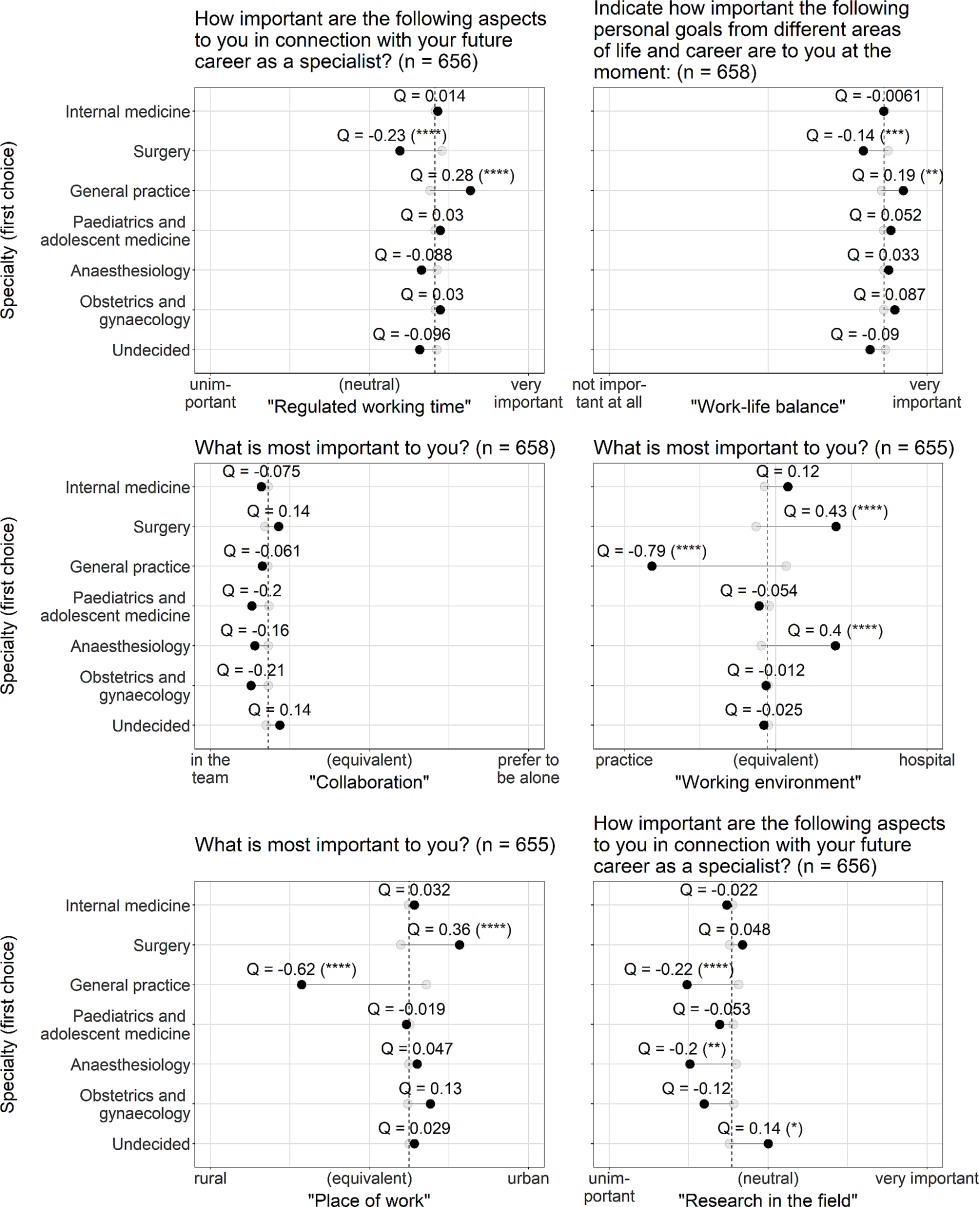
Students’ ideas concerning future practice/working conditions as medical professionals and influence on career choice. For a detailed description of the lollipop plot, see Fig. 2

Odds ratios (OR) were normalized using Yule’s Q formula [31], which transforms the OR values to a range between − 1 and 1, which is more intuitive in regard to the many different scales used. A value of 0 indicates no association between variables. Lollipop plots (Figs. 2, 3 and 4) represent the mean values of each relevant factor for either the group of students selecting the distinct specialty or not (shown in different colour). By displaying Q values for each mean difference, one can directly assess the quantity of association. A dashed line represents the mean value of the whole cohort for each factor.

Significant OR/ANOVA results are indicated as * (*p* < 0.05), ** (*p* < 0.01), *** (*p* < 0.001), and **** (*p* < 0.0001). Given the fact, that our study is explorative, we did not adjust for multiple comparisons.

### Data management, data protection, anonymity

The data protection officer of the University of Würzburg was consulted in preparation of the study. Written informed consent was obtained from all participants in compliance with the EU General Data Protection Regulation (GDPR). All data were collected and processed in an anonymised manner. To ensure anonymity, only the year of birth was requested instead of the complete date of birth. The collected data are currently stored by the Office of the Dean of Studies and will be deleted ten years after completion of the overall study in which the survey was conducted.

## Results

### Descriptive statistics of respondents

A total of 683 out of 1406 students completed the survey, of whom 481 were female (70%), resulting in an overall response rate of 49%. The characteristics of respondents are summarised in Table 1. On average, participants were 24 years old (standard deviation of 4 years). The majority of students were German nationals (637, 93%, multiple answers were allowed).

**Table 1:** Characteristics of the surveyed semesters

Semester	Group	Total *N*	Responses *n*	Survey period	Mean age	Females	Origin		
					(years)		DE	EU	non-EU
1	1	167	76	17.05. − 18.06.2021	22.0	58	70	5	7
1	2	177	90	02.11. − 25.11.2021	20.5	70	83	3	6
1	3	164	84	14.11. − 12.12.2022	20.9	54	76	5	5
5	1	157	69	06.05. − 01.06.2021	23.3	43^#^	66	2	2
5	2	165	74	10.01. − 23.03.2023	22.9	52^#^	72	4	0
10	1	143	66	26.05. − 01.07.2021	26.0	41*	62	3	3
12	1	153	84	13.09. − 16.10.2021	27.3	58^#^	82	2	5
12	2	153	80	01.03. − 14.04.2022	28.0	59	72	4	5
12	3	127	60	14.02. − 03.04.2023	26.8	46	54	5	4
	Total	1,406	683			481	637	33	37

*: one student declared their gender as “diverse”; ^#^: no gender statement by one student

### Student preferences of specialty and changes during medical studies

Figure 1A presents the ranking of first-choice specialties based on study phases and gender differences. Out of 35 options, only seven exceeded 5%: internal medicine (15%), surgery (14%), general practice (13%), paediatric and adolescent medicine (11%), anaesthesiology (9%), obstetrics and gynaecology (7%), and “undecided” (6%) – representing 75% of all students in the sample. As students progressed through their studies, the level of uncertainty declined (ANOVA for full sample: *p* < 0.0001) with more students opting for disciplines other than surgery (*p* < 0.05). These effects remained significant when conducting a separate analysis specifically for female students. Furthermore, their preference for paediatrics and adolescent medicine increased throughout the curriculum (*p* < 0.05). On the other hand, men’s’ specialty preferences remained more or less stable with only negligible change within the choice of surgery.

**Fig. 5:**
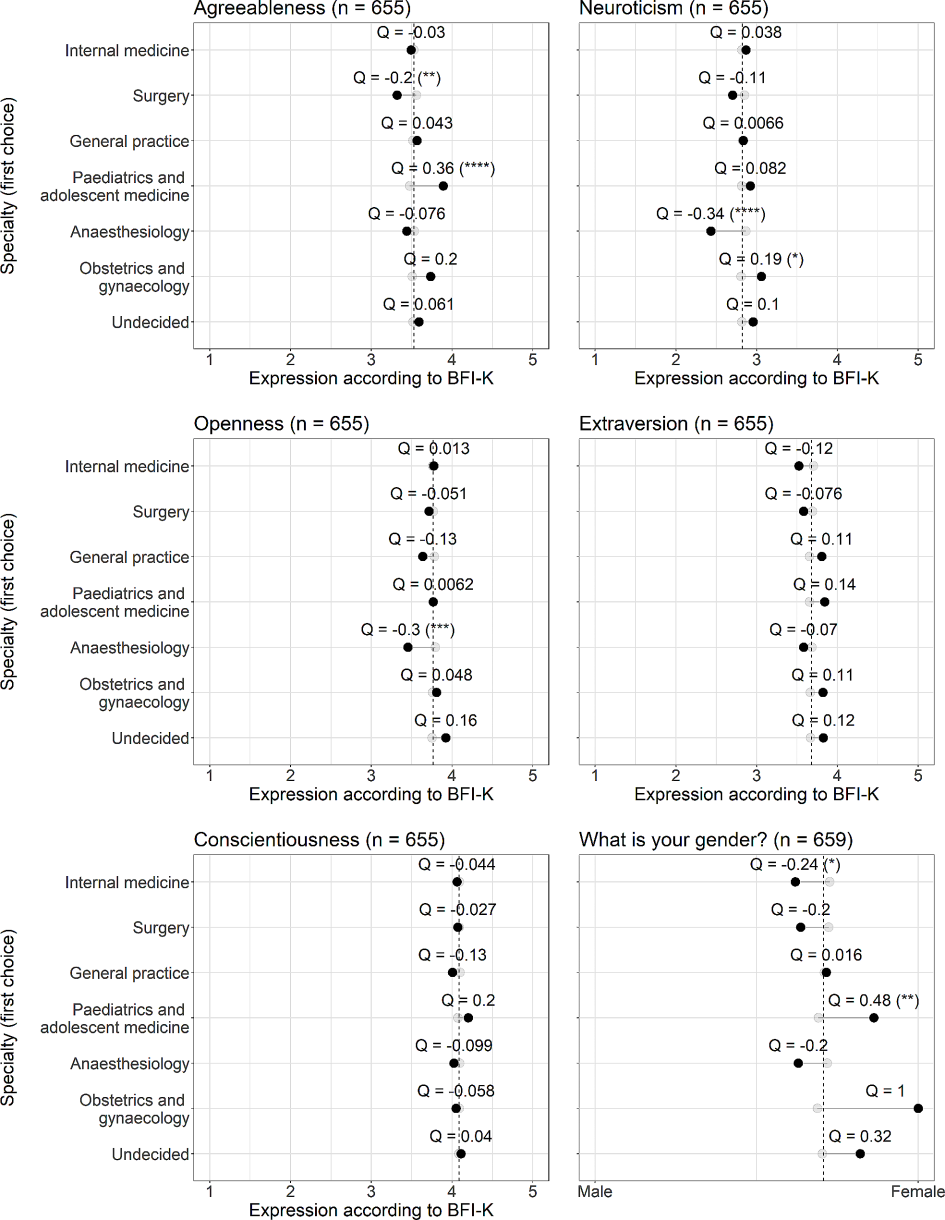
Impact of personality factors (Big Five) and gender on the choice of specialty. For a detailed description of the lollipop plot, see Fig. 2

Throughout the course of the study, the gender distribution (see Fig. 1B) mostly remained unchanged with the proportion of women exceeding two-thirds (70% in the entire sample). Exceptions were found using ANOVA for specialties in distinct study phases: internal medicine (chosen by more male students at the outset), surgery (chosen by more male students during the clinical phase), paediatric and adolescent medicine (chosen by more female students during the clinical phase), and obstetrics and gynaecology (chosen only by female students throughout all study phases). OR analyses of gender effects on the choice of specialty for the entire cohort are depicted in Fig. 5, which reveal significant variations for internal medicine and paediatric and adolescent medicine.

The establishment of specialty choices in students was significantly associated with various aspects related to their course of studies (Fig. 2). Students who reported having made their choice prior to the start of their studies were more likely to choose surgery or paediatric and adolescent medicine. Conversely, not having established the choice before starting medical school was associated with obstetrics and gynaecology, as well as being undecided. Clinical clerkships in a specific (freely chosen) specialty aligned with the career choice, except for general practice, which was a mandatory clerkship. The final practical year did not have any impact on career choice. However, the frequency with which the option “undecided” was chosen substantially decreased at such an advanced stage of medical school. Higher proportions of support from institutions or foundations, either financial or otherwise, were significantly associated with the choice of internal medicine as a specialty. Conversely, a lower proportion of institutional support was associated with the choice of surgery. Working as a student assistant was significantly associated with choosing paediatric and adolescent medicine. Not having started a doctoral thesis was highly associated with being indecisive as a student.

### Admission characteristics, motives to study medicine, and role models

Figure 3 provides a summary of students’ admission characteristics, their motives, and role models prior to medical school. Students who graduated from high school in small towns or rural areas, with lower grades in their university entrance qualification (mean difference: 0.2 to 0.3), or had completed a higher number of vocational training placements were statistically more likely to choose anaesthesiology and general practice as their specialty. Higher grades in university entrance qualification (mean difference: 0.2) were significantly associated with choosing obstetrics and gynaecology or paediatric and adolescent medicine. Students opting for paediatric and adolescent medicine were less influenced by prestige in their motivation to study medicine, while those choosing internal medicine were more strongly influenced. A greater presence of positive role models in general practice increased the likelihood of selecting that specialty. A scarcity of positive role models was associated with a higher likelihood of choosing surgery. Positive role models within the family had less influence on choosing anaesthesiology or paediatrics and adolescent medicine.

### Ideas concerning future practice/working conditions

Figure 4 illustrates students’ ideas regarding their future practice of medicine. High ratings of regulated working time were significantly associated with the choice of general practice, as well as the importance of work-life balance. On the other hand, lower ratings of these two items were associated with surgery. Surgery, along with anaesthesiology, associated more strongly with the hospital environment, whereas general practice was more associated with work in a practice setting. Furthermore, surgery was found to be more closely associated with urban areas, while general practice had a stronger association with rural areas. Students choosing general practice and anaesthesiology placed less emphasis on research. Undecided students rated research higher. Of note, no significant association was found between the choice of specialty and collaboration, which was rated at high levels towards “in a team” across all specialties.

### Personality traits (big five) and gender

Figure 5 portrays the influence of personality factors and gender on the first choice of students. Higher levels of agreeableness were associated with a greater likelihood of selecting paediatric and adolescent medicine as first choice, while surgery was attributed to individuals with lower agreeableness. Conversely, lower levels of neuroticism and openness were linked to choosing anaesthesiology, and lower levels of extraversion were associated with opting for internal medicine. Higher levels of neuroticism were found to be linked with the choice of obstetrics and gynaecology. No significant association was observed between conscientiousness and the selected specialty, which was generally rated at very high levels. Notably, male students proved to be significantly more likely to choose internal medicine (62% male vs. 38% female), whereas paediatric and adolescent medicine was chosen predominantly by female students (86% female vs. 14% male); only female students chose obstetrics and gynaecology, which was shown to be statistically insignificant on OR and ANOVA analyses (see also Fig. 1).

## Discussion

### Student preferences of specialty and changes during medical studies

The concept of our survey was specifically to probe factors that influence students in their first choice of specialty.

The top specialties chosen by the respondents across all semesters of the medical degree course in Würzburg were internal medicine, surgery, general practice, and paediatric and adolescent medicine, ranging between 10 and 15%. Anaesthesiology, and obstetrics and gynaecology followed closely behind. Not surprisingly, a few students (6%) were undecided with ever decreasing proportions during the progression of their degree courses. Even though international comparisons should be approached with caution, students across various medical schools in the USA also expressed high interest in paediatrics (20%) and surgery (15%) [32]. In accordance with our findings, most students amended their specialty choices, regardless of initial interest. In the aforementioned study, a significant proportion of 30% remained in primary care, that is, in general practice and paediatric and adolescent medicine.

We noticed a significant decline in surgery as students progressed through their degree course. However, analysing student groups divided by gender, this effect did not remain significant, except for the gender distribution during the clinical phase and a general difference between study phases for the female group (see Fig. 1). The observation of low student interest in pursuing a surgical career upon completion of medical school is not uncommon and has been noted previously [33]. However, a longitudinal study from another German medical school (Jena) could not confirm this [34]. Therefore, further investigation, especially with longitudinally tracked students, is necessary. Students’ career choices and changes are most likely influenced by their educational experiences. Students discover different specialties, interact with healthcare professionals, engage in clinical experiences and learn from role models, thus shaping their interests and priorities. In our study, we found significant results indicating a strong alignment between career choices and the clinical clerkships that students freely select during their studies. This alignment reflects the regulations in place, according to which students are required to complete a minimum of four clinical clerkships, each one lasting one month. Clerkship in general practice is mandatory for all students; thus, there was no association between career choices and this clerkship. In the literature, clinical clerkships in surgery [35] and exposure to rural places of work with primary care are commonly known to affect the attitude of medical students [36, 37]. Some authors report the importance of engaging students as early as possible even before starting clinical placements [38]. In our study, we were able to observe additional effects related to the curriculum, such as support, work experience as student assistants, and engagement in research activities. The concept of career decision-making is a dynamic and evolving process, which can be modulated when there is awareness. Therefore, promotion programmes in Germany that include elective courses in general practice during the final practical year, mentorship during the clinical phase of studies, and social events in specific areas, as well as offers for finding a scientific project have already proven to be effective in generating interest [39–43].

### Admission characteristics, motives to study medicine, and role models

Admission characteristics, such as background and high school grades of the university entrance qualification (the German Abitur), have an impact on the choice of specialty as well. In our study, we demonstrated that students who originated from small towns at the time of graduating from high school, or had lower grades in the university entrance qualification, or had completed vocational training placements were statistically more likely to choose anaesthesiology and general practice. There is evidence that rural origin is a major predictor of medical students intending to work in primary care [44]. The willingness to study medicine and pursue a career is also often fostered by previous training in closely related areas of healthcare, such as nursing, physiotherapy, or paramedics [45]. In Germany, the factor of vocational training comes into play, as it is used as a selection criterion in certain admission quotas, especially for candidates not categorized as top performers in high-school examinations [23, 46]. In particular, the introduction of the rural doctor quota requires a vocational qualification as a condition for a state-bound selection procedure. The aforementioned aspects well reflect the socio-demographic background and training prerequisites, which can influence students’ perceptions of certain specialties and their likelihood of choosing them [47, 48]. The findings of our study support the notion of socio-cultural factors in shaping career aspirations.

### Ideas concerning future practice/working conditions

We also demonstrated that students’ preferences for workstyle and location play a role in career choices. Not surprisingly, there were contrasting views on regulated working hours and work-life balance between general practice and surgery, with students who chose the surgical discipline rating the two items lower. This is consistent with the literature, as personal values, such as the desire of a favourable work-life balance, are known to influence specialty choice among medical students in favour of non-surgical disciplines [49]. A restrictive statement must however be made that, although the differences were statistically significant, students with surgical preferences still highly rate the working desires of the current generation of young physicians. Moreover, we observed that students have a good understanding of specialisation being bound to work environments (hospital versus practice) and the urban-rural distribution of facilities. In fact, surgery and anaesthesiology require hospital settings with advanced technological equipment, which can be found in larger hospitals or high-volume centres mostly situated in urban locations [50, 51]. General practice, on the other hand, serves the purpose of providing primary care, with a focus on outpatient settings and a broad distribution across the country, including rural areas. In summary, our exploration of students’ ideas about their future medical practice aligns with the growing recognition of work-life balance and available healthcare infrastructure. Previous research has highlighted the impact of working conditions, job satisfaction, and adherence of career choices [52, 53]. Our findings underscore the need for healthcare institutions and policymakers to create supportive and desirable work environments, to improve the transparency of decision-making processes.

On the interpersonal level, several studies have revealed that medical students’ career decisions are influenced by role models and mentoring opportunities [54, 55]. In our study, positive role models were associated with general practice. In contrast, role models within the family were even negatively associated with anaesthesiology, and paediatric and adolescent medicine. Positive as well as negative interactions with practicing physicians or other personal experiences with specific specialties can inspire and motivate students to pursue or avoid particular specialties [56]. This highlights the need for effective mentorship programmes and exposure to diverse role models to promote interest and engagement in various specialties [57, 58].

### Personality traits (big five) and gender

Numerous studies into the influence of personality characteristics on choice of specialty have been published in the medical education literature [59–62]. Personality traits have been shown to play a role in career decision-making, with certain traits being particularly aligned [63, 64]. Our findings indicate, both in positive and negative aspects, that personality factors such as agreeableness, neuroticism, and openness influence the choice of specialty among medical students. Agreeable students tended to choose paediatric and adolescent medicine, while neuroticism was associated with a preference for obstetrics and gynaecology. However, lower levels of neuroticism and openness were associated with the choice of anaesthesiology. Recently, the predilection of clinical medicine was also found to be associated with agreeableness and openness [65]. In an earlier study, higher values of neuroticism were associated with the preference of obstetrics and gynaecology and even connected to higher values for females in comparison with males [66]. Interestingly, we did not find any significant personality traits associated with the preferences for further specialisation in general practice.

Gender differences in specialty choices have indeed been widely observed, with women known to opt for general practice, paediatrics, or obstetrics and gynaecology [67–69]. Our study confirmed that female students predominantly chose paediatrics and adolescent medicine. Contrary to existing data, we could not confirm any gender preference regarding general practice or surgery. Only during the clinical phase significantly higher interest of male students for surgery could be confirmed. However, any findings of gender bias underscore the importance of promoting diversity and addressing gender disparities in medical specialties [70, 71].

### Limitations

The survey was conducted at a single and traditionally oriented medical school in Germany, which may limit the range of student backgrounds, experiences, and perspectives included. Factors specific to the institution, such as the curriculum, focus on specialties, or institutional culture, may have influenced students’ specialty preferences and career choices. The characteristics and preferences of students in different regions or countries may vary, and therefore caution should be exercised when extrapolating the results. Furthermore, the sample size and composition impacts the representativeness of our findings. It is possible that certain subgroups of students were underrepresented or not adequately captured. The data collected relied on self-reporting measures, which are subject to potential biases, such as social desirability bias or recall bias. Students’ responses are known to be influenced by their perception of what is expected or desired, leading to over- or under-reporting of certain factors influencing their career choices. Finally, the study employed a cross-sectional design, capturing data at a specific point in time.

## Conclusions

The findings of our study offer valuable insights into the factors that influence specialty choice among medical students. By identifying and addressing the factors, we will be able to explore strategies that support and enhance the decision-making process as needed. It is also particularly important to understand when the decision is taken during medical studies, to ensure effective education policies and strategies. No doubt, training and accompanying programmes have to be created to illustrate the possibilities, resources, and support services available [2]. By adopting this approach, comprehensive patient care can be provided across different specialties, with the goal of delivering high-quality and equitable healthcare to individuals of all needs and populations.

Further research is needed to develop deeper insights to monitor student preferences as a longitudinal approach. Exploring the impact of educational interventions, mentoring programmes, and career guidance initiatives could support students in making well-informed choices aligned with their interests and those of healthcare system needs.

### Electronic supplementary material

Below is the link to the electronic supplementary material.

Supplementary Material 1

## Data Availability

The datasets used and analysed during the study are available upon reasonable request from the corresponding author.
