# Supplementary material for: Factors associated with medical students’ career choice in different specialties: a multiple cross-sectional questionnaire study at a German medical school
Source: BMC Med Educ. 2024 Jul 24;24:798. doi: 10.1186/s12909-024-05751-1 (PMC11270969; doi:10.1186/s12909-024-05751-1)
Supplement: Supplementary file 1 — Supplementary Material 1 [file 12909_2024_5751_MOESM1_ESM.docx]

Supplemental material: survey excerpt (items relevant to the current study)

The option "not specified" (original German: “keine Angabe”) was available for all scale-based items because responses were mandatory to ensure complete feedback. In contrast, answering open-ended questions was voluntary.

For single and multiple-choice items, all options are displayed below the respective question against a grey background. For Likert scales and semantic differentials, the instructions and scale labels are presented with orange background before listing the items (as in the questionnaire). Section titles and introductory remarks are included for completeness and are displayed with a blue background.

| Nr. | Type | German (original) | English (translated) |
| --- | --- | --- | --- |
| 2 | Section title | Demografische Angaben | Demographic Information |
| 2.1 | Open question | Bitte nennen Sie Ihr Geburtsjahr (z. B. 1998): | Please state your year of birth (e. g. 1998) |
| 2.2 | Single choice | Welches Geschlecht haben Sie? | What is your gender? |
|  | Option 1 | Weiblich | Female |
|  | Option 2 | Männlich | Male |
|  | Option 3 | Divers | Diverse |
| 2.3 | Multiple choice | In welchem Land haben Sie Staatsangehörigkeit(en)?  *(Mehrfachnennungen möglich)* | In which country (or countries) do you hold citizenship?  *(Multiple responses possible)* |
|  | Option 1 | in Deutschland | in Germany |
|  | Option 2 | in einem anderen europäischen Land | in another European country |
|  | Option 3 | in einem außereuropäischen Land | in a non-European country |
| 2.4 | Single choice | Welche Größe hatte Ihr Wohnort zur Zeit Ihres Schulabschlusses? | What was the size of your place of residence at the time of your school graduation? |
|  | Option 1 | Landgemeinde (unter 5.000 Einwohner) | Rural community (fewer than 5,000 residents) |
|  | Option 2 | kleine Kleinstadt (ab 5.000 bis unter 10.000 Einwohner) | Small town (from 5,000 to fewer than 10,000 residents) |
|  | Option 3 | große Kleinstadt (ab 10.000 Einwohner bis unter 20.000 Einwohner) | Large town (from 10,000 to fewer than 20,000 residents) |
|  | Option 4 | kleine Mittelstadt (ab 20.000 bis unter 50.000 Einwohner) | Small city (from 20,000 to fewer than 50,000 residents) |
|  | Option 5 | große Mittelstadt  (ab 50.000 bis  unter 100.000  Einwohner) | Medium-sized city (from 50,000 to fewer than 100,000 residents) |
|  | Option 6 | Kleinere Großstadt (ab 100.000 Einwohner) | Small major city (from 100,000 residents) |
|  | Option 7 | große Großstadt (ab 500.000 Einwohner) | Large major city (from 500,000 residents) |
| 3 | Section title | Zulassungsquoten und –kriterien | Admission criteria and quotas |
|  | Section introduction | Im Folgenden möchten wir in Erfahrung bringen, wie Ihr Zugang zum Medizinstudium war. | In the following section, we would like to learn about your access to medical school. |
| 3.6 | Single choice | Bitte nennen Sie Ihre Abiturnote/Durchschnittsnote der Hochschulzugangsberechtigung | Please indicate your Abitur grade / average grade of your university entrance qualification |
|  | Best option | 0,9 | 0.9 |
|  | Worst option | 4,0 | 4.0 |
|  | Options between | Increments of 0.1 | |
| 3.12 | Single choice | Wie viele Berufsausbildungen haben Sie abgeschlossen? | How many vocational training courses have you completed? |
|  | Option 1 | keine | 0 |
|  | Option 2 | 1 | 1 |
|  | Option 3 | 2 | 2 |
|  | Option 4 | mehr als 2 | more than 2 |
| 4 | Section title | Interessen, Vorbilder und Studienwahl | Interests, Role Models and Choice of Study |
|  | Introduction for five-step Likert scale items | Bitte beurteilen Sie die nachfolgenden Motive, inwiefern sie Ihre Studienwahl beeinflusst haben: | Please evaluate the following motives and indicate how they influenced your choice of study: |
|  | Labelling of scale level 1 | überhaupt nicht | not at all |
|  | Labelling of scale level 5 | sehr stark | very strongly |
| 4.18 | five-step Likert scale item | Hohes Prestige des Arztberufes | High prestige of the medical profession |
| 4.21 | Multiple choice | Gab/gibt es aus folgenden Bereichen positive Erfahrungen/Vorbilder, die Ihre Studienwahl beeinflusst haben?  *(Mehrfachnennungen möglich)* | Were/are there positive experiences or role models from the following areas that influenced your choice of study?  *(Multiple responses possible)* |
|  | Option 1 | Familie | Family |
|  | Option 2 | Freunde | Friends |
|  | Option 3 | Allgemeinmedizin | General practice |
|  | Option 4 | Andere Fachgebiete/Disziplinen in Niederlassung/Praxen | Other specialties/disciplines in private practice/clinics |
|  | Option 5 | Krankenhaus | Hospital |
|  | Option 6 | Medien (z. B. Bücher, Serien) | Media (e. g. books, series) |
|  | Option 7 | Sonstige | Other |
|  | Option 8 | Keine Vorbilder | No role models |
| 5 | Section title | Soziales | Social |
|  | Section introduction | Im Folgenden fragen wir nach Faktoren bezüglich Motivation und Ausrichtung Ihres Lebens an sich. | In the following, we ask about factors related to the motivation and overall direction of your life in general. |
|  | Introduction for seven-step Likert scale items | Bitte geben Sie an, wie wichtig Ihnen momentan die folgenden persönlichen Ziele aus verschiedenen Lebens- und Berufsbereichen sind: | Please indicate how important the following personal goals from various life and career areas are to you at the moment: |
|  | Labelling of scale level 1 | überhaupt nicht | not at all |
|  | Labelling of scale level 7 | sehr stark | very strongly |
| 5.7 | seven-step Likert scale item | Vereinbarkeit von Beruf und Privatleben | Work-life balance |
| 5.13 | Single choice | Haben Sie während des Studiums eine Förderung durch eine Stiftung oder Institution erhalten? | Did you receive any support, financial or otherwise, from a foundation or institution during your studies? |
|  | Option 1 | Ja | Yes |
|  | Option 2 | Nein | No |
| 6 | Section title | Studienverlauf | Course of studies |
|  | Section introduction | Im Folgenden geht es um Ihren Studienverlauf. | The following section is about your course of study. |
| 6.6 | Single choice | Haben Sie eine Tätigkeit als studentische Hilfskraft ausgeübt oder üben diese weiterhin aus? | Have you worked as a student assistant or are you currently working as one? |
|  | Option 1 | Ja | Yes |
|  | Option 2 | Nein | No |
| 6.11 and 6.13 | Multiple choice  (underlined part only in 6.11; bold face part only in 6.13) | Welches Gebiet/welche Gebiete der Facharzt- und Schwerpunktkompetenzen hat/haben Ihre stationäre/n/**ambulante/n** Famulatur/en umfasst?  *(Mehrfachnennungen möglich.)* | Which area(s) of specialty and subspecialty competencies did your inpatient/**outpatient** clinical clerkship(s) cover?  *(Multiple responses possible.)* |
|  | Option 1 | Allgemeinmedizin | General practice |
|  | Option 2 | Anästhesiologie | Anaesthesiology |
|  | Option 3 | Arbeitsmedizin | Occupational medicine |
|  | Option 4 | Augenheilkunde | Ophthalmology |
|  | Option 5 | Chirurgie | Surgery |
|  | Option 6 | Frauenheilkunde und Geburtshilfe | Obstetrics and gynaecology |
|  | Option 7 | Hals-Nasen-Ohren-Heilkunde | Otorhinolaryngology |
|  | Option 8 | Haut- und Geschlechtskrankheiten | Dermatology |
|  | Option 9 | Humangenetik | Human genetics |
|  | Option 10 | Hygiene und Umweltmedizin | Hygiene and environmental medicine |
|  | Option 11 | Innere Medizin | Internal medicine |
|  | Option 12 | Kinder und Jugend-Medizin | Paediatric and adolescent medicine |
|  | Option 13 | Kinder und Jugendpsychiatrie und –psychotherapie | Child and adolescent psychiatry and psychotherapy |
|  | Option 14 | Mikrobiologie, Virologie und Infektionsepidemiologie | Microbiology, virology and infection epidemiology |
|  | Option 15 | Mund-Kiefer-Gesichts-Chirurgie | Oral and maxillofacial surgery |
|  | Option 16 | Neurochirurgie | Neurosurgery |
|  | Option 17 | Neurologie | Neurology |
|  | Option 18 | Nuklearmedizin | Nuclear medicine |
|  | Option 19 | Öffentliches Gesundheitswesen | Public health |
|  | Option 20 | Pathologie | Pathology |
|  | Option 21 | Pharmakologie | Pharmacology |
|  | Option 22 | Physikalische und Rehabilitative Medizin | Physical  and rehabilitation medicine |
|  | Option 23 | Psychiatrie und Psychotherapie | Psychiatry and psychotherapy |
|  | Option 24 | Psychosomatische Medizin und Psychotherapie | Psychosomatic medicine and psychotherapy |
|  | Option 25 | Radiologie | Radiology |
|  | Option 26 | Rechtsmedizin | Forensic medicine |
|  | Option 27 | Strahlentherapie | Radiation Therapy |
|  | Option 28 | Transfusionsmedizin | Transfusion medicine |
|  | Option 29 | Unterstützung der Bekämpfung der Corona-Epidemie | Support in combatting the coronavirus epidemic |
|  | Option 30 | Urologie | Urology |
| 7 | Section title | Promotion | Doctorate |
| 7.1 | Single choice | Haben Sie eine Promotion (Doktorarbeit) angefangen? | Have you started a doctoral thesis (dissertation)? |
|  | Option 1 | Ja | Yes |
|  | Option 2 | Nein | No |
| 8 | Section title | Facharztorientierung | Choice of specialty |
|  | Section introduction | Die folgenden Fragen betreffen Ihre Facharztorientierung zum gegenwärtigen Zeitpunkt. | The following questions pertain to your current orientation towards specialty training. |
|  | Introduction for five-step Likert scale items | Wie wichtig sind Ihnen die folgenden Aspekte im Zusammenhang mit Ihrer späteren fachärztlichen Tätigkeit? | How important are the following aspects to you in connection with your future career as a specialist? |
|  | Labelling of scale level 1 | überhaupt nicht | not at all |
|  | Labelling of scale level 5 | sehr stark | very strongly |
| 8.8 | five-step Likert scale item | Geregelte Arbeitszeit | Regulated working time |
| 8.10 | five-step Likert scale item | Forschung im Fach | Research in the field |
|  | Introduction to semantic differential | Im Folgenden finden Sie meist gegensätzliche Antwortmöglichkeiten zu einem bestimmten Aspekt der ärztlichen Tätigkeit. Bitte wählen Sie aus, was Ihnen am wichtigsten ist.  Hinweis: Stehen Sie den Paaren indifferent gegenüber oder bewerten Sie beide als gleichwertig, so wählen Sie bitte die Mitte. | In the following, you will find mostly opposing response options regarding a medical practice. Please choose what is most important to you.  Note: If you feel indifferent toward the pairs or evaluate both as equally important, please select the middle option |
|  | Neutral label for all items | gleichwertig | equivalent |
| 8.20 | Semantic differential | Arbeitsumgebung | Working Environment |
|  | Option 1 | Klinik | Hospital |
|  | Option 2 | Praxis | Practice |
| 8.21 | Semantic differential | Arbeitsort | Workplace |
|  | Option 1 | Stadt | Urban |
|  | Option 2 | Land | Rural |
| 8.27 | Semantic differential | Zusammenarbeit | Collaboration |
|  | Option 1 | lieber allein | prefer to be alone |
|  | Option 2 | im Team | in a team |
| 8.36 | Single choice | Welche Spezialisierungsrichtung ist derzeit Ihre erste Wahl? | Which specialization is currently your first choice? |
|  | Option 1 | Allgemeinmedizin | General practice |
|  | Option 2 | Anästhesiologie | Anaesthesiology |
|  | Option 3 | Anatomie | Anatomy |
|  | Option 4 | Arbeitsmedizin | Occupational medicine |
|  | Option 5 | Augenheilkunde | Ophthalmology |
|  | Option 6 | Biochemie | Biochemistry |
|  | Option 7 | Chirurgie | Surgery |
|  | Option 8 | Frauenheilkunde und Geburtshilfe | Obstetrics and gynaecology |
|  | Option 9 | Hals-Nasen-Ohren-Heilkunde | Otorhinolaryngology |
|  | Option 10 | Haut- und Geschlechtskrankheiten | Dermatology |
|  | Option 11 | Humangenetik | Human genetics |
|  | Option 12 | Hygiene und Umweltmedizin | Hygiene and environmental medicine |
|  | Option 13 | Innere Medizin | Internal medicine |
|  | Option 14 | Kinder und Jugend-Medizin | Paediatric and adolescent medicine |
|  | Option 15 | Kinder und Jugendpsychiatrie und –psychotherapie | Child and adolescent psychiatry and psychotherapy |
|  | Option 16 | Laboratoriumsmedizin | Laboratory medicine |
|  | Option 17 | Mikrobiologie, Virologie und Infektionsepidemiologie | Microbiology, virology and infection epidemiology |
|  | Option 18 | Mund-Kiefer-Gesichts-Chirurgie | Oral and maxillofacial surgery |
|  | Option 19 | Neurochirurgie | Neurosurgery |
|  | Option 20 | Neurologie | Neurology |
|  | Option 21 | Nuklearmedizin | Nuclear medicine |
|  | Option 22 | Öffentliches Gesundheitswesen | Public health |
|  | Option 23 | Pathologie | Pathology |
|  | Option 24 | Pharmakologie | Pharmacology |
|  | Option 25 | Physikalische und Rehabilitative Medizin | Physical  and rehabilitation medicine |
|  | Option 26 | Physiologie | Physiology |
|  | Option 27 | Psychiatrie und Psychotherapie | Psychiatry and psychotherapy |
|  | Option 28 | Psychosomatische Medizin und Psychotherapie | Psychosomatic medicine and psychotherapy |
|  | Option 29 | Radiologie | Radiology |
|  | Option 30 | Rechtsmedizin | Forensic medicine |
|  | Option 31 | Strahlentherapie | Radiation Therapy |
|  | Option 32 | Transfusionsmedizin | Transfusion medicine |
|  | Option 33 | Urologie | Urology |
|  | Option 34 | Keine Weiterbildung | No further specialization |
|  | Option 35 | Unentschlossen | Undecided |
| 8.37 | Multiple choice | Wann hat sich Ihre Facharztwahl gefestigt bzw. herausgebildet?  *(Mehrfachnennungen möglich.)* | When did you establish or substantiate your choice of specialty?  *(Multiple responses possible.)* |
|  | Option 1 | Vor Studienbeginn | Before starting my degree |
|  | Option 2 | Während des Studiums gefestigt | Consolidated during my degree |
|  | Option 3 | Erst während des Studiums  aufgekommen | Only emerged during my degree |
|  | Option 4 | Während des Blockpraktikums | During the clinical rotations |
|  | Option 5 | Während der Famulatur | During the clinical clerkship |
|  | Option 6 | Während des Praktischen Jahres | During the practical year |
|  | Option 7 | Während der Promotion | During the doctorate |
|  | Option 8 | Noch unentschlossen | Still undecided |
| 10 | Section title | Persönlichkeit | Personality |
|  | Section introduction | Nun interessieren uns Ihre persönlichen Einstellungen. Bitte geben Sie bei den folgenden Aussagen jeweils an, inwieweit diese Aussage grundsätzlich auf Sie zutrifft. Bei Ihren Antworten gibt es weder „richtige“ noch „falsche“, weder „gute“ noch „schlechte“ Antworten. Bitte treffen Sie Ihre Auswahl möglichst spontan. | Now we are interested in your personal attitudes. For each of the following statements, please indicate to what extent each of the following statements generally applies to you. There are no "right" or "wrong", "good" or "bad" answers. Please make your selection as spontaneously as possible. |
|  | Introduction for five-step Likert scale items | Inwieweit treffen die folgenden Aussagen auf Sie persönlich zu? Ich… | To what extent do the following statements apply to you personally? I… |
|  | Labelling of scale level 1 | sehr unzutreffend | very inapplicable |
|  | Labelling of scale level 2 | eher unzutreffend | rather inapplicable |
|  | Labelling of scale level 3 | weder noch | neither nor |
|  | Labelling of scale level 4 | eher zutreffend | rather applicable |
|  | Labelling of scale level 5 | sehr zutreffend | very applicable |
| 10.1 | Likert scale item | ... bin eher zurückhaltend. | ... am rather reserved. |
| 10.2 | Likert scale item | ... neige dazu, andere zu kritisieren. | … tend to criticize others. |
| 10.3 | Likert scale item | … erledige Aufgaben gründlich. | ... complete tasks thoroughly. |
| 10.4 | Likert scale item | … werde leicht deprimiert, niedergeschlagen. | … easily get depressed, feel down. |
| 10.5 | Likert scale item | … bin vielseitig interessiert. | ... have a wide range of interests. |
| 10.6 | Likert scale item | … bin begeisterungsfähig und kann andere leicht mitreißen. | … am enthusiastic and can easily inspire others. |
| 10.7 | Likert scale item | … schenke anderen leicht Vertrauen, glaube an das Gute im Menschen. | … trust others easily, believe in the good in people. |
| 10.8 | Likert scale item | … bin bequem, neige zur Faulheit. | …am lazy, tend to be idle. |
| 10.9 | Likert scale item | … bin entspannt, lasse mich durch Stress nicht aus der Ruhe bringen. | … am relaxed, do not get easily stressed. |
| 10.10 | Likert scale item | .... bin tiefsinnig, denke gerne über Sachen nach. | ... am thoughtful, like to deliberate. |
| 10.11 | Likert scale item | … bin eher der „stille Typ“, wortkarg. | … am more the "quiet type", laconic. |
| 10.12 | Likert scale item | … kann mich kalt und distanziert verhalten. | … can behave in a cold and distant manner. |
| 10.13 | Likert scale item | … bin tüchtig und arbeite flott. | … am efficient and work quickly. |
| 10.14 | Likert scale item | … mache mir viele Sorgen. | … worry a lot. |
| 10.15 | Likert scale item | … habe eine aktive Vorstellungskraft, bin phantasievoll. | ... have an active imagination. |
| 10.16 | Likert scale item | … gehe aus mir heraus, bin gesellig. | … am outgoing, sociable. |
| 10.17 | Likert scale item | … kann mich schroff und abweisend anderen gegenüber verhalten. | … can behave brusquely and dismissively towards others. |
| 10.18 | Likert scale item | … mache Pläne und führe sie auch durch. | ... make plans and carry them out. |
| 10.19 | Likert scale item | .... werde leicht nervös und unsicher. | … easily become nervous and insecure. |
| 10.20 | Likert scale item | … schätze künstlerische und ästhetische Eindrücke. | … appreciate artistic and aesthetic impressions. |
| 10.21 | Likert scale item | … habe nur wenig künstlerisches Interesse. | ... have little interest in the arts. |
